# A clinically relevant pulse treatment generates a bortezomib-resistant myeloma cell line that lacks proteasome mutations and is sensitive to Bcl-2 inhibitor venetoclax

**DOI:** 10.1038/s41598-022-17239-3

**Published:** 2022-07-27

**Authors:** Sondra L. Downey-Kopyscinski, Sriraja Srinivasa, Alexei F. Kisselev

**Affiliations:** 1 Department of Molecular and Systems Biology, and Norris Cotton Cancer Center, Geisel School of Medicine, Dartmouth College, Hanover, NH USA; 2 Department of Drug Discovery and Development, Harrison College of Pharmacy, Auburn University, PRB, 720 S. Donahue Dr., Auburn, AL 36849 USA; 3 Present Address: SLDK-Rancho Biosciences, San Diego, CA USA

**Keywords:** Cancer therapy, Haematological cancer, Cancer models

## Abstract

Proteasome inhibitors bortezomib and carfilzomib are the backbones of treatments of multiple myeloma, which remains incurable despite many recent advances. With many patients relapsing despite high initial response rates to proteasome inhibitor-containing regimens, it is critical to understand the process of acquired resistance. In vitro generated resistant cell lines are important tools in this process. The majority of previously developed bortezomib-resistant cell lines bear mutations in the proteasome PSMB5 sites, the prime target of bortezomib and carfilzomib, which are rarely observed in patients. Here we present a novel bortezomib-resistant derivative of the KMS-12-BM multiple myeloma cell line, KMS-12-BM-BPR. Unlike previously published bortezomib-resistant cell lines, it was created using clinically relevant twice-weekly pulse treatments with bortezomib instead of continuous incubation. It does not contain mutations in the PSMB5 site and retains its sensitivity to carfilzomib. Reduced load on proteasome due to decreased protein synthesis appears to be the main cause of resistance. In addition, KMS-12-BM-BPR cells are more sensitive to Bcl-2 inhibitor venetoclax. Overall, this study demonstrates the feasibility of creating a proteasome inhibitor resistant myeloma cell lines by using clinically relevant pulse treatments and provides a novel model of acquired resistance.

## Introduction

Proteasome inhibitors bortezomib (Btz), carfilzomib (Cfz) and ixazomib (Ixz) are the backbones of the treatment of multiple myeloma (MM). The proteasome is a large proteolytic complex that degrades abnormal and misfolded proteins and thus plays a key role in the protein quality control and maintenance of protein hemostasis in every mammalian cell. Multiple myeloma cells secrete large amounts of immunoglobulins, creating an enormous load on protein quality control machinery, making them exquisitely sensitive to proteasome inhibitors^1–5^. Btz partially inhibits proteasome by blocking its β5 sites, which are responsible for the chymotrypsin-like activity, and to a lesser extent, β1 sites, which are responsible for the caspase-like activity. Cfz is a more specific and potent inhibitor of the chymotrypsin-like activity^6^. Such partial inhibition causes a build-up of abnormal proteins specifically in myeloma cells, ultimately leading to apoptosis.

While the initial response rates of proteasome inhibitor-containing combinations approach 90%^7,8^, patients eventually relapse and become resistant to all FDA-approved treatments. A better understanding of the mechanism of resistance to proteasome inhibition is necessary to improve treatment for resistant and refractory patients. Resistant cell lines created by the in vitro exposure to the low concentrations of the agent are important tools widely used to gain an understanding of the mechanism of resistance. The majority of Btz-resistant MM cell lines developed by this approach display mutations in the β5 sites^9–14^, which reduce Btz binding. However, these mutations are rarely detected in relapsed patients^15–18^, which raises questions about the clinical relevance of these models. Most of these cell lines were created by continuously culturing cells in Btz, which does not accurately mimic the clinical pharmacokinetics of the drug administered twice weekly as a bolus^19,20^. Here we describe the development and initial characterization of a novel Btz-resistant KMS-12-BM cell line that lacks mutations in the β5 site and was created by twice-weekly one-hour pulse treatments cells with increasing concentrations of Btz.

## Results

### Creation of a resistant KMS-12-BM-BPR sub-line

**Figure 1:**
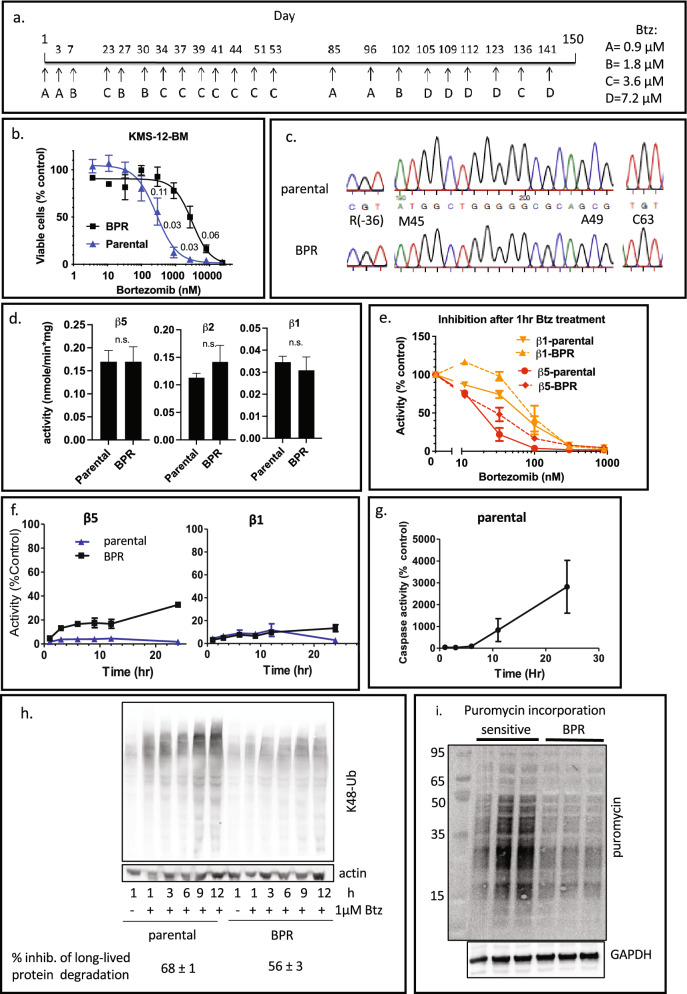
Development and characterization of Btz-resistant KMS-12-BM-BPR cells. (**a**) The timeline of the creation of the KMS-12-BM-BPR cells by 1 h pulse treatment with increasing concentrations of Btz. (**b**) The viability of resistant and parental cells was compared by the Alamar Blue mitochondrial dye conversion assay 48 h after 1 h pulse-treatment of cells, n = 4. Numbers on the graph are p-values. Results of continuous treatment are presented on Fig. S1a. (**c**) Selected sequencing data demonstrating the lack of mutations described in the literature Btz-resistant. The sequencing data for all exons of the gene is presented on Fig. S2. (**d**) β5 peptidase activities of three active sites were measured in lysates of untreated cells with Suc-LLVY-amc (β5), Ac-nLPnLD-amc (β1), Ac-RLR-amc (β2), n = 3. (**e**) Inhibition of proteasome in cells was measured immediately after 1 h Btz treatment using Proteasome Glo assay, n = 2. (**f**) Recovery of activity after 1 h pulse treatment with 1 µM Btz was determined by Proteasome Glo, n = 3. (**g**) The parental KMS-12-BM cells were treated with 1 μM Btz for 1 h, then cultured in a drug-free media, and assayed for Caspase 3/7 activity. (**h**) Accumulation of K48-linked ubiquitylated proteins at different times after 1 h treatment with 1 μM Btz. Inhibition of proteasomal degradation of long-lived protein was measured in cells treated with 1 μM Btz and compared with mock-treated controls; n = 3. See Fig. 3c for a biological replicate. (**i**) The incorporation of chain terminator puromycin in the nascent polypeptide chain was analyzed by western. Biological replicates are shown. Uncropped images are presented in Fig. S3a.

To develop a Btz-resistant cell line using clinically relevant pulse treatment, we treated KMS-12-BM cells twice weekly with 1 h pulses of Btz. As resistance developed, we increased the dose stepwise from 900 nM to 7.2 µM Btz (Fig. 1a) over the course of 6 months, until we derived a sub-line that was approximately tenfold more resistant to a 1 h-pulse (Fig. 1b) and continuous Btz treatment (Fig. S1a). We named it KMS-12-BM-BPR, where BPR stands for “bortezomib-pulse resistant”. For brevity, we will also refer to it as BPR. This sub-line did not require culturing in the presence of Btz to maintain resistance and grew at the same rate as the parental line (Fig. S1b). Thus, it is feasible to develop Btz-resistant cell lines using clinically relevant pulse treatments.

### KMS-12-BPR cells line does not have mutations and does not overexpress proteasomes

We tested, by Sanger sequencing of genomic DNA, whether the BPR cells have a mutation in or around Btz binding pocket of the PSMB5 (β5c) subunit, which reduced Btz affinity to its prime target, as in the majority of previously developed Btz-resistant cells^9–11^. We did not find any specific mutations in the resistant cells (Fig. 1c and Fig. S2). Some of the previously reported resistant cells overexpressed the β5 subunits^9^, resulting in an increase in proteasome activity. However, there were no significant changes in the activities of any of the proteasome three catalytic subunits (Fig. 1d).

Consistent with a lack of mutations, 1 h pulse treatment with Btz caused similar inhibition of β5 (i.e., the combined activity of β5c and β5i) and β1 (i.e., combined activity of β1c and β1i) activities in the parental cells and the resistant subclone (Fig. 1e). Although there were small differences in inhibition of β5 activity at sub-toxic Btz concentrations, inhibition was the same in cells treated with 1 μM Btz, which causes the largest differences in viability. Thus, resistance in the BPR sub-line is not due to decreased affinity of Btz to the active sites.

### Faster recovery of the proteasome activity after pulse treatment could contribute to resistance

We measured the proteasome activity in the cells over time after a pulse treatment with 1 μM Btz, which causes maximal differences in sensitivity (Fig. 1b). The resistant cells have slightly faster recovery of the β5 activity, but not the β1 activity. 20% of β5 activity recovered 4-6 h after treatment in resistant cells (Fig. 1f), which is before the onset of apoptosis (Fig. 1g) raising the possibility that differences in recovery rates may contribute to resistance.

To determine whether different recovery rates translate into differences in protein degradation, we measured the degradation of long-lived proteins in a pulse-chase experiment. We also measured the accumulation of undegraded K48-linked ubiquitylated proteins, most of which are derived from undegraded nascent polypeptides^21^. After 1 h treatment, both approaches revealed a modest difference between sensitive and resistant cell lines, with resistant cells slightly less susceptible to inhibition (Fig. 1h). Despite modest initial differences, sensitive cells accumulated substantially more ubiquitylated proteins over time. The difference was notable at 6 h and preceded the onset of apoptosis, which was detected only at 12 h (Fig. 1g), and therefore is more likely to be the cause of cell death and not the consequence of decreased proteasome activity in dying cells. The decreased accumulation of ubiquitylated proteins in resistant cells could be caused by the activation of deubiquitylating enzymes. However, we did not detect any activation of deubiquitylating enzymes using the ubiquitin-methyl ester activity-based probe^22^ (Fig. S1c). Therefore, the difference in the degradation rates is the most likely cause of resistance.

The sensitivity of myeloma cells to proteasome inhibitors is defined by the ratio of the load on the proteasome, i.e. amount of proteins degraded, to proteasome capacity, i.e. amount of active proteasomes^1–5^. Since proteasome activity in the resistant cells was similar (Fig. 1d), increased capacity cannot account for resistance, suggesting that lower load is the primary cause. The majority of ubiquitylated proteins that accumulate upon treatment of cells with proteasome inhibitors are derived from nascent polypeptides^21^; therefore higher protein synthesis rates should result in a higher load on the proteasome. We used puromycin incorporation assay^23^ to measure rates of protein synthesis and found that resistant cells incorporate substantially less puromycin than the sensitive ones indicating slower protein synthesis rates (Fig. 1i). Thus, Btz resistance in KMS-12-BM-BPR cells correlates with the decreased load on the proteasome due to a lower protein synthesis rate.

### Cfz and marizomib (Mzb), but not Ixz overcome Btz resistance

**Figure 2:**
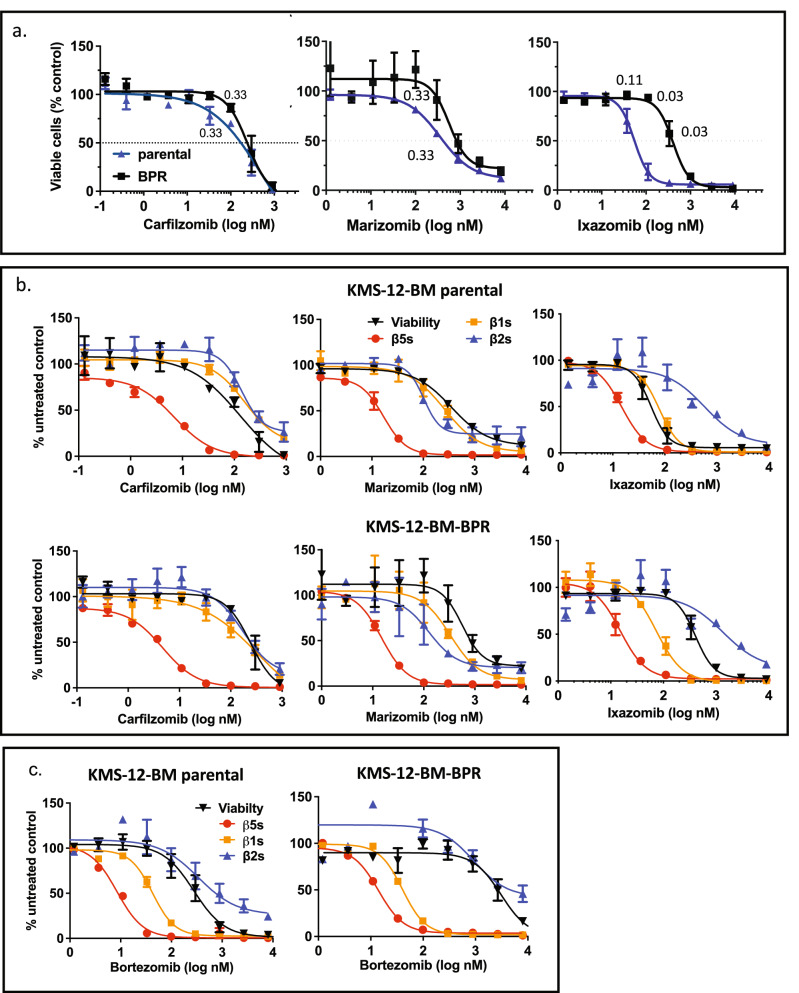
Cfz and Mzb but no Ixz overcome resistance to Btz. (**a**) Cells were treated with Cfz (n = 2) and Mzb (n = 2) for 1 h, and Alamar Blue assay was performed 48 h later. Cells were treated with Ixz continuously (n = 4) because Ixz is reversible and persists in patient blood much longer than other inhibitors^44,45^. The numbers on the graphs are p-values. Continuous treatment with Cfz also reversed resistance (Fig. S1d). (**b**,**c**) Inhibition of each active site was measured with Proteasome-Glo immediately after 1 h treatment and overlayed on viability data from (**a**), (**b**) or Fig. 1b (**c**); n = 2.

We next tested if other proteasome inhibitors could overcome Btz resistance in the KMS-12-BM-BPR line (Fig. 2). We observed that Cfz, which is approved by the FDA for the treatment of multiple myeloma, and marizomib (Mzb, salinosporamide A, NPI-0052), a natural product undergoing clinical trials in multiple myeloma and glioblastoma, can overcome resistance (Fig. 2a and Fig. S1d). However, Ixz, an orally bioavailable FDA-approved analog of Btz, did not. Unlike Cfz and Mzb, Btz and Ixz do not inhibit proteasome β2 sites, responsible for the trypsin-like activity. Although all four inhibitors blocked the β5 sites with similar potency, we found that decrease of viability in Cfz and Mzb-treated cells coincided with co-inhibition of β1 and β2 sites (Fig. 2b). In comparison, Ixz and Btz inhibit β2 activity at higher concentrations than β1 (Fig. 2b,c). Thus, inhibition of β2 sites appears critical to overcoming the Btz-resistance of KMS-12-BM BPR cells.

### Increasing proteasome inhibition by targeting the β2 subunits can overcome Btz resistance

**Figure 3:**
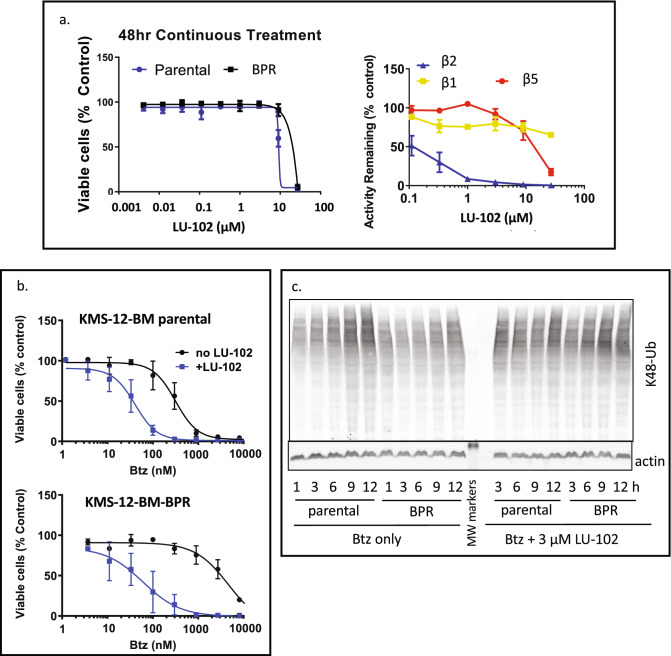
LU-102 overcomes resistance in BPR cells. (**a**) Effect of a single agent LU-102 treatments. Cells were treated continuously with LU-102 for 48 h before viability was determined using Alamar Blue; in a parallel experiment, activity was measured with Proteasome Glo 2 h after treatment; n = 2. (**b**) Cells were treated with Btz for 1 h when it was replaced with 3 µM LU-102 for 48 h, when Alamar Blue assay was performed, n = 2–3. (**c**) Cells were treated with 1 µM Btz for 1 h and then allowed to recover in media in the presence or absence of 3 µM LU-102. Cell lysates were analyzed by westerns. Uncropped images are presented in Fig. S3b.

The ability of inhibitors that co-inhibit β2 sites to overcome Btz resistance is consistent with an expectation of the load-to-capacity hypothesis that resistant cells, which withstand partial proteasome inhibition due to their low load-to-capacity ratio, will succumb to proteasome inhibitors that further decrease proteasome capacity by blocking additional active sites. We previously found that β2-specific inhibitor LU-102 is a potent sensitizer of myeloma and solid tumor cells to Btz and Cfz, and that it overcomes acquired resistance to these agents^24–27^. To confirm the importance of β2 sites for Btz resistance in the BPR line, we tested whether LU-102 restores Btz sensitivity. We found that specific blocking of β2 sites by LU-102 did not affect cell viability of parental and resistant cells (Fig. 3a). When 1 h Btz treatment was followed by subsequent treatment with sub-toxic, β2-specific concentrations of LU-102, both cell lines were sensitized to Btz (Fig. 3b). However, sensitization was more robust in resistant cells. Co-treatment with LU-102 sensitized resistant cells to a wider range of Btz concentrations. The effect of LU-102 on the viability of resistant cells was most noticeable at 1 μM Btz, where the addition of LU-102 restored the Btz sensitivity of KMS-12-BM-BPR cells to the level of the wild type. At this concentration, co-treatment with LU-102 accelerated the accumulation of ubiquitylated proteins (Fig. 3c), confirming that LU-102 acts through inhibition of the proteasome. Thus, inhibition of β2 sites overcomes resistance to Btz in KMS-12-BM-BPR cells.

### KMS-12-BM-BPR cells are highly sensitive to Bcl-2 inhibition

**Figure 4:**
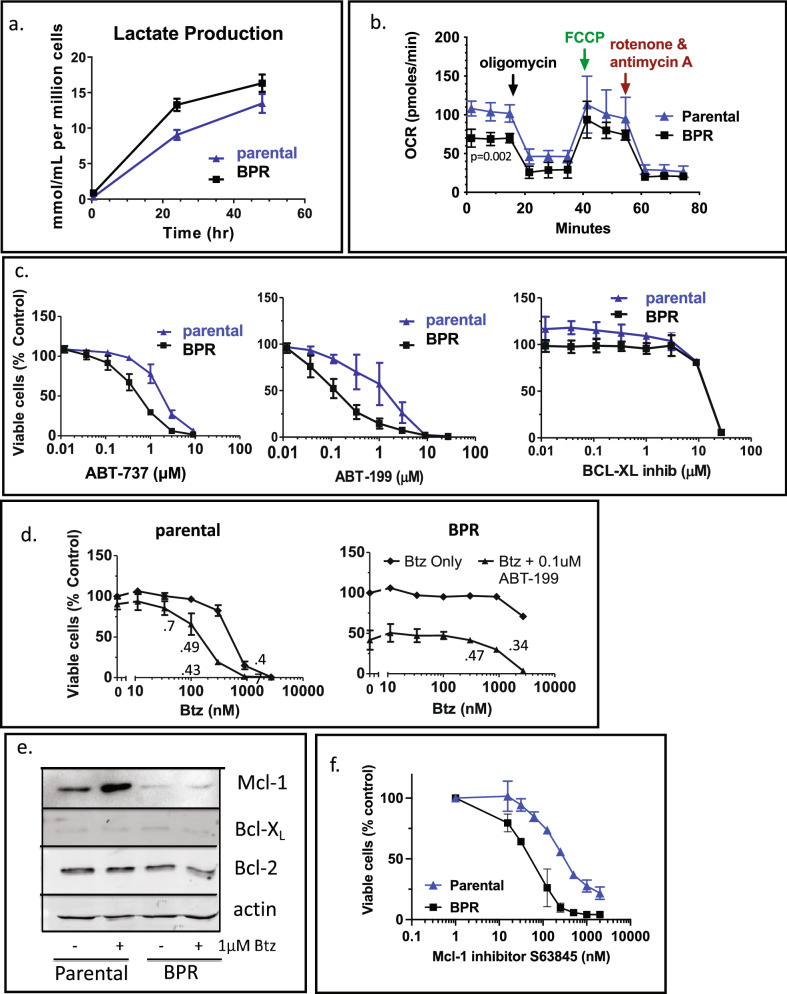
KMS-12-BM-PPR are more sensitive to BCL-2 inhibition than the parental cells. (**a**) Lactate production was measured over time and normalized for cell count. (**b**) Oxygen consumption rate was measured before and after exposure to the inhibitors of the electron transport chain and oxidative phosphorylation. (**c**) Cells were treated continuously for 48 h with ABT-737 before being assayed for viability with Alamar Blue (n = 2). (**c**) BPR cells are more sensitive to a Bcl-2-specific inhibitor ABT-199, but not Bcl-X_L_ specific inhibitor. Left, cells were treated for 48 h with ABT-199 (left) or Bcl-X_L_ inhibitor (A-1155463) before viability was determined by Alamar Blue (n = 2–3). (**d**) Cells were treated with Btz for 1 h and then with ABT-199 for 47 h before viability was determined by Alamar Blue (n = 2–4). The numbers on the graph are combination indexes (CI). (**e**) Expression of Bcl-2 family member proteins was determined by westerns following 1 h pulse treatment with Btz and overnight recovery. Uncropped images are presented on Fig. S3c. (**f**) Cells were treated for 48 h with Mcl-1 inhibitor S63845 before viability was determined by Alamar Blue (n = 2).

We noticed that the resistant cells consistently turned phenol-red-containing media orange and yellow (indicative of a pH change) at a faster rate than their parental cells. We confirmed increased lactate production (Fig. 4a), and reduced rates of basal mitochondrial respiration (Fig. 4b). KMS-12-BM cells bear t(11;14) translocation. According to the literature, myeloma cells bearing t(11;14) translocation have lower oxygen consumption rates than myeloma cells without this translocation^28^ and are more sensitive to Bcl-2 inhibitor ABT-199 (venetoclax)^29,30^. KMS-12-BM, however, is not the most venetoclax-sensitive myeloma line^29^. Therefore, we decided to investigate whether KMS-12-BM-BPR are more sensitive to Bcl-2 inhibitors than the parental cells.

We observed increased sensitivity of the resistant cells to the dual Bcl-2/Bcl-X_L_ inhibitor, ABT-737 (Fig. 4b), and to Bcl-2 specific inhibitor ABT-199 (venetoclax, Fig. 4c) but not to the BCL-X_L_ specific inhibitor A-1155463^31^ (Fig. 4c). When ABT-199 followed a 1 h Btz treatment, the combination was synergistic in both cell lines (Fig. 4d). The sensitivity of myeloma cells to venetoclax depends on Bcl-2:Mcl-1 and Bcl-2:Bcl-X_L_ expression ratios^30^. Therefore, to determine the mechanism of increased Bcl-2 dependence, we examined the expression of Bcl-2 members and found that Mcl-1 expression was much lower in the resistant cells, but Bcl-2 was not overexpressed in the resistant cells (Fig. 4e). Bcl-X_L_ expression was low in both lines (Fig. 4e). Surprisingly, reduced Mcl-1 expression did not reduce Mcl-1 dependence as BPR cells were more sensitive to Mcl-1 inhibitor S63845 (Fig. 4d). Thus, acquired resistance to Btz sensitizes KMS-12-BM-BPR cells to Bcl-2 and Mcl-1 inhibitors.

## Discussion

With the creation of the KMS-12-BM-BPR cell line, we demonstrate that it is possible to isolate proteasome inhibitor-resistant myeloma cells using a clinically relevant pulse treatment of established myeloma cell lines with Btz. Several properties of this line indicate that this approach generates a clinically relevant model. Like the majority of clinical cases, and in contrast to the majority of Btz-resistant cell lines described in the literature, the KMS-12-BM-BPR line does not have mutations in the β5 site. Decreased immunoglobulin production as the result of de-differentiation was described in myeloma-resistant patient samples^32^, and although the KMS-12-BM line does not secrete immunoglobulins, we found dramatically decreased protein synthesis rate in the KMS-12-BM-BPR line. De-differentiated myeloma cells express B-cell markers, and it was recently reported that expression of B-cell markers correlates with venetoclax sensitivity in myeloma cells^29^. Our observation that decreased protein synthesis in resistant cells leads to increased sensitivity to venetoclax is similar to that finding. Our results also correlate with a previously reported ability of venetoclax to overcome Btz resistance and synergize with Btz in a fraction of patients bearing t(11;14) translocation^33,34^. Similar to venetoclax responders in the clinical studies^33^, the KMS-12-BM-BPR line had a higher Bcl-2 to Mcl-1 ratio. This ability of Bcl-2 inhibitors to overcome Btz resistance has not been described in the in vitro generated cell lines, however, none of them bear the t(11;14) translocation which sensitizes myeloma cells to Bcl-2 inhibitors.

Several previous studies have reported metabolic adaptations in Btz-resistant cells, and some Btz-resistant cells with increased mitochondrial metabolism are also sensitive to Bcl-2 inhibitors^35^. Three studies have found that mitochondrial metabolism and oxidative phosphorylation are up-regulated in cell lines^35–37^ and myeloma patient cells^18^. On the other hand, Bajpai et al. found that venetoclax-sensitive myeloma cells bearing t(11;14) translocation have lower oxygen consumption rates than myeloma cells that do not bear this translocation^28^. Conversely, B-cell lymphoma cell lines with acquired venetoclax resistance had higher oxygen consumption rates and higher expression of Mcl-1^38^. Our observation that KMS-12-BM-BPR cells have lower oxygen consumption rate, lower expression of Mcl-1, and are more sensitive to venetoclax, is consistent with the latter observation from the literature. KMS-12-BM-BPR appears to resemble Btz-resistant RPMI-8226 cells, which were reported to have increased lactate production, in addition to the higher activity of pentose phosphate and serine biosynthesis pathways^39^. Exploring metabolic changes in the resistant cell was not the goal of our study, and it remains to be determined whether increased production of lactate and decreased oxygen consumption are required to maintain a resistant phenotype or are by-products of resistance development.

Cfz overcomes resistance to Btz as it does in many Btz-refractory patients. We found that Cfz and Mzb, which produce more potent inhibition of β2 sites than Btz, overcome resistance, and that co-treatment with β2-specific inhibitor also overcomes resistance. On the other hand, KMS-12-BM-BPR cells were cross-resistant to Ixz, which does not inhibit β2 sites. This observation is also in agreement with our previous findings that co-inhibition of β2 sites overcomes Btz resistance in primary cells from myeloma patients^26^, and that combined β2 and β5 inhibition produces a stronger anti-neoplastic effect than combined β1 and β5 inhibition^25^. This data also agrees with clinical observations that the efficacy of Cfz in MM seems to correlate with the co-inhibition of β2i sites^40^.

In summary, we have demonstrated that it is feasible to generate resistant cells by a clinically relevant pulse exposure to Btz, and have generated a model Btz-resistant cell line that bears a t(11;14) translocation.

## Material and methods

### Cell lines

KMS-12-BM cell line was provided by Takemi Otsuki (Kawasaki Medical School, Japan). All cell culture was performed using RPMI-1640 media supplemented with 10% fetal bovine serum (FBS), penicillin (100 μg/mL), streptomycin (100 units/mL), and anti-mycoplasma antibiotic Plasmocin (1.5 μg/mL, Invivogen, San Diego, CA). KMS-12-BM-BPR subline was generated by repeated exposure of KMS-12-BM to a 1 h-pulses of increasing concentration of Btz, ranging from 0.9 to 7.2 μM over 6 months (Fig. 1a). The line was then maintained in a regular media in the absence of Btz without loss of resistance. The parental and the resistant lines were authenticated by STR profiling.

### Inhibitors, viability, and cell-based activity assays

Btz and Cfz were purchased from LC laboratories. Ixz was provided by Millennium Pharmaceuticals. Mzb was the kind gift of Dr. Bradley Moore*.* LU-102 was synthesized as previously described and provided by Dr. Herman Overkleeft^27^. Rotenone, carbonyl cyanide 4-(trifluoromethoxy)phenylhydrazone (FCCP), oligomycin, and antimycin A used in oxygen consumption assay were acquired from Sigma. All compounds were dissolved in DMSO and then further diluted in tissue culture media. Viable cells were assessed with Alamar Blue mitochondrial dye conversion assay as described^41^. Caspase 3/7 activity was determined using Apo-One Homogeneous Caspase-3/7 Assay (Promega). Proteasome activity in cultures was measured using Proteasome Glo assay (Promega) that contains site-specific luminogenic substrates Suc-LLVY-amino-luciferin (aLuc, β5 sites), Z-nLPnLD-aLuc (β1 sites), and Boc-LRR-aLuc (β2 sites), which are specifically cleaved by proteasome in MM cells^41,42^. Activity in extracts was measured using a standard Suc-LLVY-4-amino-methyl coumarin (amc), Ac-nLPnLD-amc and Ac-RLR-amc substrates as described^43^. Lactate production was measured with a Lactate Assay Kit (Sigma). Oxygen consumption was determined on a Seahorse XFP metabolic analyzer. Cells were plated on Seahorse PDL mini plates at a density of 10^5^ cells/well in Seahorse XF RPMI media, containing 1% FBS, on an evening before the experiment. Oxygen consumption rate was recorded before and after consecutive addition (as indicated by arrows) of 1.5 μM oligomycin, 2 μM FCCP, 0.5 μM rotenone and 0.5 μM antimycin A.

### Western blotting

Frozen cell pellets were lysed in 50 mM Tris–HCl, pH 7.5, 10% glycerol, 5 mM MgCl_2_, 1 mM EDTA, and 0.5% CHAPS. Protein content of lysates was determined by Pierce 660 nm protein assay reagent (ThermoFisher Scientific) and used to normalize gel loading. Lysates were run on NuPAGE gels (Invitrogen) using either MOPS or MES (Fig. 4e and Fig. S3d) running buffer before being transferred to a PDVF membrane, which was then blocked with Odyssey Blocking Buffer (LiCor), or 5% fat-free milk. Following antibodies were used: anti-K48-linkage Specific Polyubiquitin (D9D5, Cell Signaling, #8081), anti-actin (Abcam ACTN05(C4), or Cell Signaling 8H10D10 Cat #3700), anti-Bcl-X_L_ (Cell Signaling #2764), anti-Bcl-2 (Dako, # M0877), and anti-Mcl-1 (BD Pharmigen, # 559027), anti-Puromycin (clone 12D10, EMD Millipore cat #MABE343), HRP-conjugated anti-mouse IgG (Cell Signaling #7074), HRP-conjugated anti-rabbit IgG (Cell Signaling #7076), IRDye800CW labelled anti-mouse-IgG (Licor, #926-68070), Alexa680-labeled anti-rabbit-IgG (Invitrogen, #A21076). Bands were revealed using SuperSignal West Femto Maximum Sensitivity Substrate (ThermoFisher Scientific) followed by imaging using a CCD camera (GelDoc (Bio-Rad) or Azure c600 instruments) or on Odyssey scanner (LiCOR) using auto-exposure settings.

### Inhibition of protein degradation

To measure the breakdown of long-lived proteins, cultures of MM cells (1 × 10^6^ cells/mL) were pulse-labeled with 30 μCi/mL ^3^H-Leucine overnight and washed 3 times with warm chase media containing Leucine at 2.5 × normal concentration (1 mM) to remove unincorporated ^3^H-Leucine. Each suspension culture was then incubated in the chase media for 1 h to allow for the degradation of short-lived proteins. Treatment with Btz (1 μM) and Z-Leu_3_-epoxyketone (ZL3ek) was performed during this chase. ZL3ek was used at a concentration that completely inhibit all three proteasomal activities to determine the background due to non-proteasomal protein degradation. After an additional wash in a chase media to remove excess inhibitors, cells were cultured in fresh chase media for 1 h when degradation was stopped by mixing cultures with 1/10 volume of ice-cold 100% trichloroacetic acid (TCA). After 25 min incubation on ice, precipitated undegraded proteins were separated from TCA-soluble degradation products by centrifugation for 15 min at 20,000*g* at + 4 °C. Pellets of TCA-precipitated proteins were washed twice with ice-cold acetone, air-dried, dissolved in 100% TCA, and counted in a scintillation plate reader alongside supernatants from the 10% TCA precipitation step. The fraction of proteins degraded was calculated by dividing total dpm in TCA supernatant by a sum of total counts in TCA supernatant and total counts in the TCA pellet. Three technical replicates were averaged, and background (e.g., degradation in the presence of ZL3ek) was subtracted to determine proteasomal degradation and inhibition in the presence of 1 μM Btz was calculated.

### Puromycin incorporation to measure protein synthesis

The rate of protein synthesis in parental and BPR cells was compared using puromycin incorporation assay^23^. Puromycin (10 μg/mL) was added to cell culture media for 25 min, after which cells were harvested, lysed, run on 10% NuPAGE gels using MES buffer, transferred to PVDF membrane, and analyzed by western using puromycin antibody.

### Data analysis

All values shown on the graphs indicate means ± S.E.M of several biological replicates, the exact number of which (n) is indicated in the caption. Statistical significance was determined by non-parametric unpaired Mann–Whitney test using GraphPad Prism.

## Supplementary Information

Supplementary Figures.

## Data Availability

KMS-12-BM-BPR cell lines are available from the corresponding offer upon request. This study did not generate any large datasets or any novel DNA sequence data. Raw Sanger sequencing data is available from the corresponding author upon reasonable request.
