# Supplementary material for: A clinically relevant pulse treatment generates a bortezomib-resistant myeloma cell line that lacks proteasome mutations and is sensitive to Bcl-2 inhibitor venetoclax
Source: Sci Rep. 2022 Jul 27;12:12788. doi: 10.1038/s41598-022-17239-3 (PMC9329464; doi:10.1038/s41598-022-17239-3)
Supplement: Supplementary file 1 — Supplementary Figures. [file 41598_2022_17239_MOESM1_ESM.pdf]

A clinically relevant pulse-treatment generates a bortezomib-resistant myeloma cell line that lacks proteasome mutations and is sensitive to Bcl-2 inhibitor venetoclax

Sondra L. Downey-Kopyscinski[1,3], Sriraja Srinivasa[2], and Alexei F. Kisselev[2]

[1]Department of Molecular and Systems Biology, and Norris Cotton Cancer Center, Geisel School of Medicine, Dartmouth College, Hanover, NH, and [2]Department of Drug Discovery and Development, Harrison College of Pharmacy, Auburn University, Auburn, AL

Running title: a novel bortezomib-resistant myeloma cell line

[3]Present affiliations: SLDK - Rancho Biosciences, San Diego, CA

Address correspondence to: Alexei F. Kisselev, PRB, 720 S. Donahue Dr., Auburn University AL 36849.

Email: AFK0006@auburn.edu; Phone: 334-844-7356; fax 334-844-8331

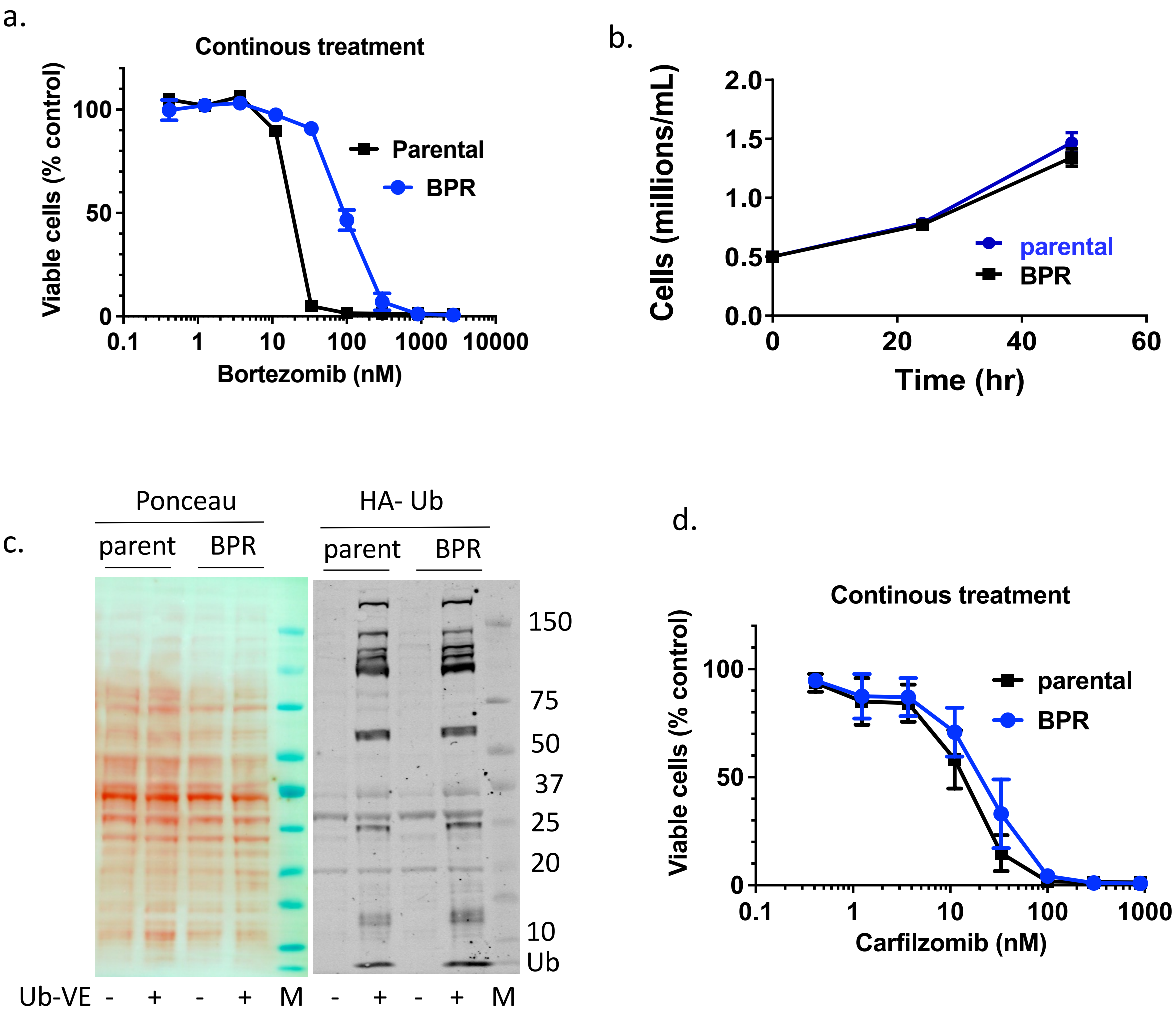

Figure S1. Analysis of KMS-12-BM-BPR cells. **a.** Cells were treated with Btz for 48hr, and viability was determined by the Alamar Blue assay; n=2. **b**. The growth rate of cells was determined by counting them using hemocytometer. **c.** Expression of de-ubiquitylating enzymes. Cell extracts were treated with 5 μM HA-tagged ubiquitin-vinyl ester (Ub-ve)[25], and analyzed by westerns. Membranes were first stained with Ponceau, and then probed with HA antibodies. Unaltered full-length images are shown. **d.** Cells were treated with Cfz for 48hr, and viability was determined by the Alamar Blue assay; n=2.

```
         Exon1
PARENTAL TCAGAATTAGTTCTTTCTGCCCACACTAGACATGGCGCTTGCCAGCGTGTTGGAGAGACCGCTACCGGTGAACCAGCGCGGGTT
ref      tcagaattagttctttctgcccacactagacatggcgcttgccagcgtgttggagagaccgctaccggtgaaccagcgcgggtt
BPR      TCAGAATTAGTTCTTTCTGCCCACACTAGACATGGCGCTTGCCAGCGTGTTGGAGAGACCGCTACCGGTGAACCAGCGCGGGTT
Protein                                  M1-start of the propeptide

TTTCGGACTTGGGGGTCGTGCAGATCTGCTGGATCTAGGTCCAGGGAGTCTCAGTGATGGTCTGAGCCTGGCCGCGCCAGGCTGGGGTGTCCC
tttcggacttgggggtcgtgcagatctgctggatctaggtccagggagtctcagtgatggtctgagcctggccgcgccaggctggggtgtccc
TTTCGGACTTGGGGGTCGTGCAGATCTGCTGGATCTAGGTCCAGGGAGTCTCAGTGATGGTCTGAGCCTGGCCGCGCCAGGCTGGGGTGTCCC
                R(-36)
                                                        Exon2
AGAAGAGCCAGGAATCGAAATGCTTCATGGAACAACCACCCTGGCCTTCAAG  TTCCGCCATGGAGTCATAGTTGCAGCTGACTCCAGGGCT
Agaagagccaggaatcgaaatgcttcatggaacaaccaccctggccttcaag  ttccgccatggagtcatagttgcagctgactccagggct
AGAAGAGCCAGGAATCGAAATGCTTCATGGAACAACCACCCTGGCCTTCAAG  TTCCGCCATGGAGTCATAGTTGCAGCTGACTCCAGGGCT
                               T  T  T  L  A  F  K     F  R  H  G  V  I  V  A  A  D  S  R  A

ACAGCGGGTGCTTACATTGCCTCCCAGACGGTGAAGAAGGTGATAGAGATCAACCCATACCTGCTAGGCACCATGGCTGGGGGCGCAGCGGAT
acagcgggtgcttacattgcctcccagacggtgaagaaggtgatagagatcaacccatacctgctaggcaccatggctgggggcgcagcggat
ACAGCGGGTGCTTACATTGCCTCCCAGACGGTGAAGAAGGTGATAGAGATCAACCCATACCTGCTAGGCACCATGGCTGGGGGCGCAGCGGAT
 T  A  G  A  Y  I  A  S  Q  T  V  K  K  V  I  E  I  N  P  Y  L  L  G  T  M45 A  G  G  A49 A

TGCAGCTTCTGGGAACGGCTGTTGGCTCGGCAATGTCGAATCTATGAGCTTCGAAATAAGGAACGCATCTCTGTAGCAGCTGCCTCCAAACTG
tgcagcttctgggaacggctgttggctcggcaatgtcgaatctatgagcttcgaaataaggaacgcatctctgtagcagctgcctccaaactg
TGCAGCTTCTGGGAACGGCTGTTGGCTCGGCAATGTCGAATCTATGAGCTTCGAAATAAGGAACGCATCTCTGTAGCAGCTGCCTCCAAACTG
 C  S  F  W  E  R  L  L  A  R  Q  C63 R  I  Y  E  L  R  N  K  E  R  I  S  V  A  A  A  S  K  L

                                                                                 Exon3
CTTGCCAACATGGTGTATCAGTACAAAGGCATGGGGCTGTCCATGGGCACCATGATCTGTGGCTGGGATAAGAGAGGCCCT  GGCCTCTAC
Cttgccaacatggtgtatcagtacaaaggcatggggctgtccatgggcaccatgatctgtggctgggataagagaggccct  ggcctctac
CTTGCCAACATGGTGTATCAGTACAAAGGCATGGGGCTGTCCATGGGCACCATGATCTGTGGCTGGGATAAGAGAGGCCCT  GGCCTCTAC
 L  A  N  M  V  Y  Q  Y  K  G  M  G  L  S  M  G  T  M  I  C  G  W  D  K  R  G  P    G  L  Y

TACGTGGACAGTGAAGGGAACCGGATTTCAGGGGCCACCTTCTCTGTAGGTTCTGGCTCTGTGTATGCATATGGGGTCATGGATCGGGGCTAT
tacgtggacagtgaagggaaccggatttcaggggccaccttctctgtaggttctggctctgtgtatgcatatggggtcatggatcggggctat
TACTTGGACAGTGAAGGGAACCGGATTTCAGGGGCCACCTTCTCTGTAGGTTCTGGCTCTGTGTATGCATATGGGGTCATGGATCGGGGCTAT
 Y  V  D  S  E  G  N  R  I  S  G  A  T  F  S  V  G  S  G  S  V  Y  A  Y  G  V  M  D  R  G  Y

TCCTATGACCTGGAAGTGGAGCAGGCCTATGATCTGGCCCGTCGAGCCATCTACCAAGCCACCTACAGAGATGCCTACTCAGGAGGTGCAGTC
tcctatgacctggaagtggagcaggcctatgatctggcccgtcgagccatctaccaagccacctacagagatgcctactcaggaggtgcagtc
TCCTATGACCTGGAAGTGGAGCAGGCCTATGATCTGGCCCGTCAAGCCATCTACCAAGCCACCTACAGAGATGCCTACTCAGGAGGTGCAGTC
 S  Y  D  L  E  V  E  Q  A  Y  D  L  A  R  R  A  I  Y  Q  A  T  Y  R  D  A  Y  S  G  G  A  V

AACCTCTACCACGTGCGGGAGGATGGCTGGATCCGAGTCTCCAGTGACAATGTGGCTGATCTACATGAGAAGTATAGTGGCTCTACCCCCTGA
aacctctaccacgtgcgggaggatggctggatccgagtctccagtgacaatgtggctgatctacatgagaagtatagtggctctacccccctga
AACCTCTACCACGTGCGGGAGGATGGCTGGATCCGAGTCTCCAGTGACAATGTGGCTGATCTACATGAGAAGTATAGTGGCTCTACCCCCTGA
 N  L  Y  H  V  R  E  D  G  W  I  R  V  S  S  D  N  V  A  D  L  H  E  K  Y  S  G  S  T  P STOP

AAGAGGGTGGATGCAGCTGCTTGTGTTTCTTGGGGTGACTGTCATTGGTAATACGGACAC
aagagggtggatgcagctgcttgtgtttcttggggtgactgtcattggtaatacggacac
AAGAGGGTGGATGCAGCTGCTTGTGTTTCTTGGGGTGACTGTCATTGGTAATACGGACAC
```

Figure S2. **Sequence of PSMB5 gene in parental KMS-12-BM cells and in Bortezomib-resistant KMS-12-BM-BPR cells.** Codon which are mutated in cell lines described in the literature are bold; residues important to catalysis are red, and Thr-1, which provided catalytic nucleophile is bold red. Residue that are involved in the formation of substrate binding pockets are highlighted: S1, green; S2, gray; S3, blue; S‘, yellow.

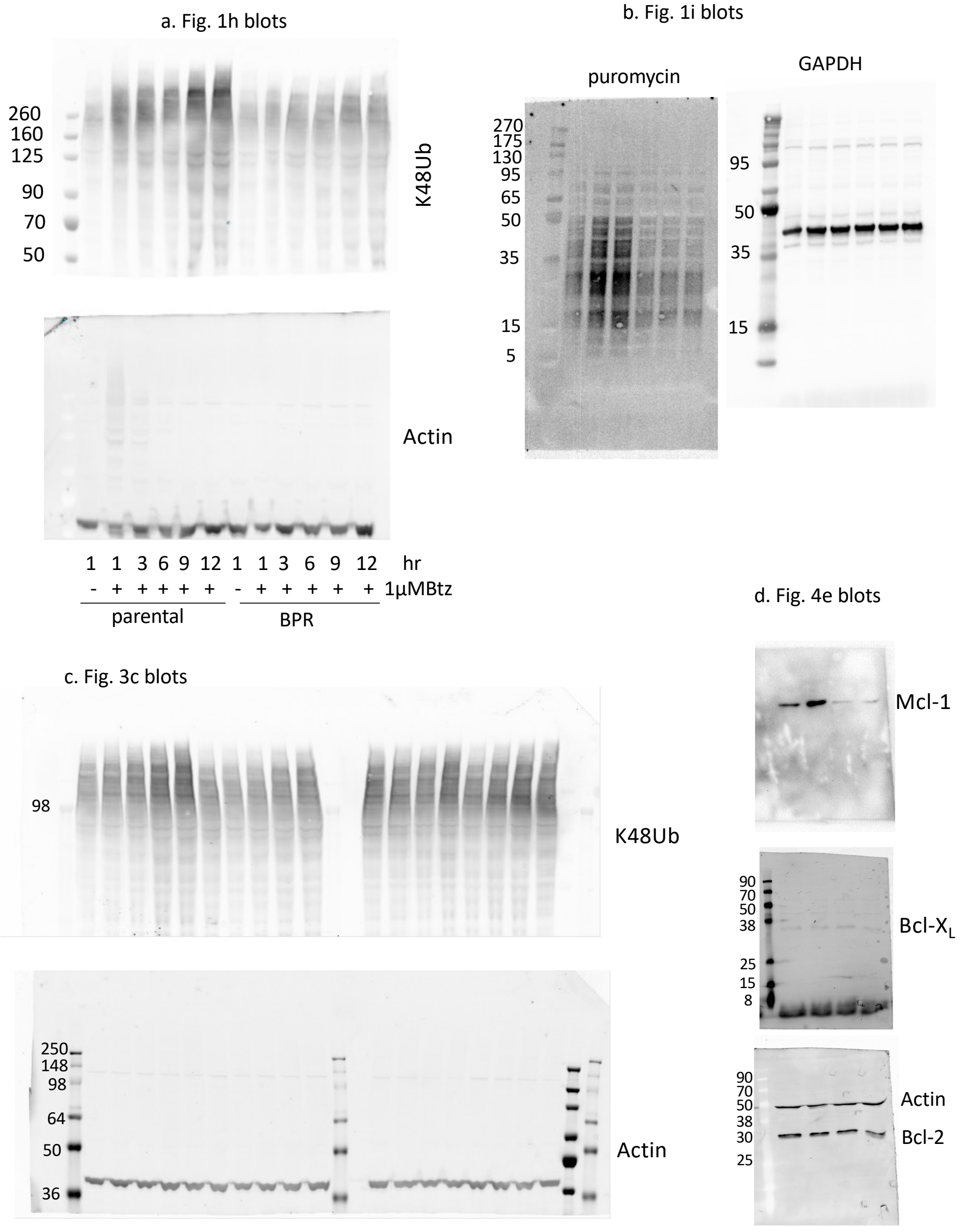

Figure S3. Uncropped images of blots. a,c Membranes were cut at ~40 kD (a) or ~30kDa (c). Bottom portions that do not contain antigens of interest were removed to save antibodies. b,d. Full length membranes are shown. Please note that gels in (d) were run using MES buffer to improve resolution of low-MW proteins.
